# C-reactive protein point of care testing in the management of acute respiratory infections in the Vietnamese primary healthcare setting – a cost benefit analysis

**DOI:** 10.1186/s13756-018-0414-1

**Published:** 2018-10-04

**Authors:** Yoel Lubell, Nga T. T. Do, Kinh V. Nguyen, Ngan T. D. Ta, Ninh T. H. Tran, Hung M. Than, Long B. Hoang, Poojan Shrestha, Rogier H. van Doorn, Behzad Nadjm, Heiman F. L. Wertheim

**Affiliations:** 1 Centre for Tropical Medicine and Global Health, Nuffield Department of Medicine, University of Oxford, Oxford, UK; 2 Mahidol Oxford Tropical Medicine Research Unit, Faculty of Tropical Medicine, Mahidol University, 420/6 Rajvithi Road, Bangkok, 10400 Thailand; 3 Oxford University Clinical Research Unit, Ha Noi, Viet Nam; 4 National Hospital for Tropical Diseases, Hanoi, Viet Nam; 5 Infectious Diseases Data Observatory, University of Oxford, Oxford, UK; 6 Department of Medical Microbiology, Radboudumc Center of Infectious Diseases, Radboudumc, Nijmegen, Netherlands

**Keywords:** C- reactive protein, Cost-benefit, Antibiotic, Primary care, Vietnam

## Abstract

**Aim:**

We assess the cost-benefit implications of C-reactive protein (CRP) testing in reducing antibiotic prescription for acute respiratory infection in Viet Nam by comparing the incremental costs of CRP testing with the economic costs of antimicrobial resistance averted due to lower antibiotic prescribing.

**Findings:**

Patients in the CRP group and the controls incurred similar costs in managing their illness, excluding the costs of the quantitative CRP tests, provided free of charge in the trial context. Assuming a unit cost of $1 per test, the incremental cost of CRP testing was $0.93 per patient. Based on a previous modelling analysis, the 20 percentage point reduction in prescribing observed in the trial implies a societal benefit of $0.82 per patient. With the low levels of adherence to the test results observed in the trial, CRP testing would not be cost-beneficial. The sensitivity analyses showed, however, that with higher adherence to test results their use would be cost-beneficial.

**Electronic supplementary material:**

The online version of this article (10.1186/s13756-018-0414-1) contains supplementary material, which is available to authorized users.

## Background

It is estimated that 80–90% of antibiotic prescription occurs in primary care, of which half are for acute respiratory infection (ARI) [1, 2]. Antibiotic use is unrestricted in Viet Nam, and prescription and sales of antibiotics for ARI is very common in- and outside primary health care settings [3] despite a predominantly viral etiology [4]. Approximately 70% of primary care patients in Viet Nam are prescribed antibiotics, and ARI is the reason for 51% of these [5]. Treatment decisions are at best based on clinical examination, which in both low and high income settings is of poor accuracy in identifying when antibiotics are required, and is often inadequately performed [3, 6, 7].

The interaction between antimicrobial consumption and antimicrobial resistance (AMR) is complex, however, it is widely accepted that safe reductions in consumption will have the desirable effect of mitigating the burden of AMR [8]. A recent modelling study estimated the economic costs of AMR per antibiotic consumed, equating with the societal gains for every course of antibiotics averted [9]. For example, in the Thai context, the consumption of a full course of beta-lactams was associated with an economic cost of $10.8 due to AMR.

Several biomarker tests have been evaluated for this purpose in the context of ARIs in primary care, and C-reactive protein (CRP) has been shown to have high discriminatory power in distinguishing between viral and bacterial infections, in the range of 85–95% sensitivity and 50–75% specificity [10–12]. A meta-analysis of clinical trials concluded that CRP tests can safely reduce antibiotic prescribing [13].

Point-of-care (PoC) CRP tests are commercially available and can be performed in primary care using capillary blood samples, with results available within minutes [14, 15]. This approach is already taken in a number of high income countries such as Norway and Sweden [16] and is recommended by Public Health England and the National Institute for Health and Clinical Excellence (NICE) [17]. In low and middle income countries (LMICs), and in Asia in particular, no such tests are in routine use and antibiotic dispensing is often unregulated or regulation is poorly enforced and resistance levels are high and increasing [18–21]. Despite the urgent need to improve antibiotic targeting in these settings, the necessary evidence on the cost-effectiveness of biomarker testing is scarce.

A randomised control trial in Viet Nam compared CRP PoC testing with routine care in the management of ARIs in primary care, finding significant reductions in antibiotic prescription without compromising patient recovery and satisfaction [22]. To determine whether the incremental cost of introducing CRP tests is economically justifiable, this needs to be compared with the societal costs of AMR the tests could avert. In this short report, we use primary cost data collected in the trial and the output of the modelling analysis of the economic costs of AMR per antibiotic consumed to perform a cost-benefit analysis of CRP testing in primary care in the Vietnamese setting.

## Methods

This cost-benefit analysis of CRP testing takes a societal perspective. Primary cost data were collected in a clinical trial on CRP testing in primary care as described in the Additional file 1: Tables S1 and S2, comparing the costs of managing ARI in the intervention group as compared with controls. The CRP readers and reagents used in the study were donated by the manufacturer, with a purchase cost per kit of approximately $3 and for a single reader $1000. If implemented at scale, CRP testing could be carried out using simple lateral flow devices that can be used by relatively untrained personnel. Such tests have been shown to be accurate and available at under $0.5 [15, 23]. By way of comparison World Health Organisation (WHO) pre-qualified lateral flow malaria rapid diagnostic tests (RDTs) are available at under $0.3. We conservatively assume here a unit cost of $1 per test, allowing for added costs for import tariffs, shipment, training, and other peripheral expenses.

The trial results showed no difference in clinical outcomes between the study arms, therefore the benefits considered here relate only to the societal costs of AMR averted due to lower prescribing. Estimates for the economic cost of AMR per antibiotic prescribed are taken from a modelling analysis where these were calculated in the US and Thai contexts [9]. The Thai costs were adjusted by a factor of 0.38 using the ratio of 2017 GDP per capita (PPP) in Viet Nam to that of Thailand (0.38 * $10.8 = $4.1). We thus assign an economic cost of AMR of $4.1 per full course of broad spectrum beta-lactams, the drug class most often prescribed in the study. The net benefit of CRP testing is calculated as

1 $$ NMBcrp=\Delta  pAB\ast cAMR-\left(\Delta  DC+ Ct\right) $$

where NMB is the net monetary benefit of CRP testing, *∆pAB* is the percentage difference in prescribing between patients in the CRP group and controls; cAMR is the cost of AMR per antibiotic consumed; *∆DC* is the difference in direct medical care as observed in the trial; and *Ct* is the direct cost of the CRP tests. A positive net monetary benefit indicates that CRP testing is cost-beneficial. All costs are assumed to be incurred at the time patients are presenting at the health centre, therefore no discounting is applied.

A three-way sensitivity analysis was carried out for key drivers of the net-monetary benefit: 1) the cost of the CRP tests, ranging from $0.5 to $3; 2) adherence to test results indicating low CRP (i.e. not prescribing antibiotics to patients with low CRP concentrations, which occurred in 64% of cases in the trial context) ranging from 50 to 100%; and 3) the economic cost of AMR, ranging from $0 to $14 per course of antibiotics, using the upper bound of the range of costs of AMR per full course of broad spectrum beta-lactam as described in Shrestha et al. (2018) [9], adjusted by the per-capita GDP ratios of Viet Nam and Thailand.

The data were analyzed and the cost-benefit analysis were run in R version 3.2.2 (R Foundation for Statistical Computing, Vienna, Austria) and using the R Color Brewer package.

## Results

### Trial primary outcome and costs

There was a 20 percentage point risk difference in prescribing on first attendance between patients in the intervention group (43%) and the controls (63%). As detailed in the Additional file 1: Table S1, the direct costs of management of ARI in the trial were $1.24 and $1.31 in the CRP group and the controls, respectively, with no significant difference (*p* = 0.28). The slight reduction in costs were explained by the lower rate of antibiotic prescribing in the intervention group.

### Modelled cost-benefit outcomes

**Fig. 1:**
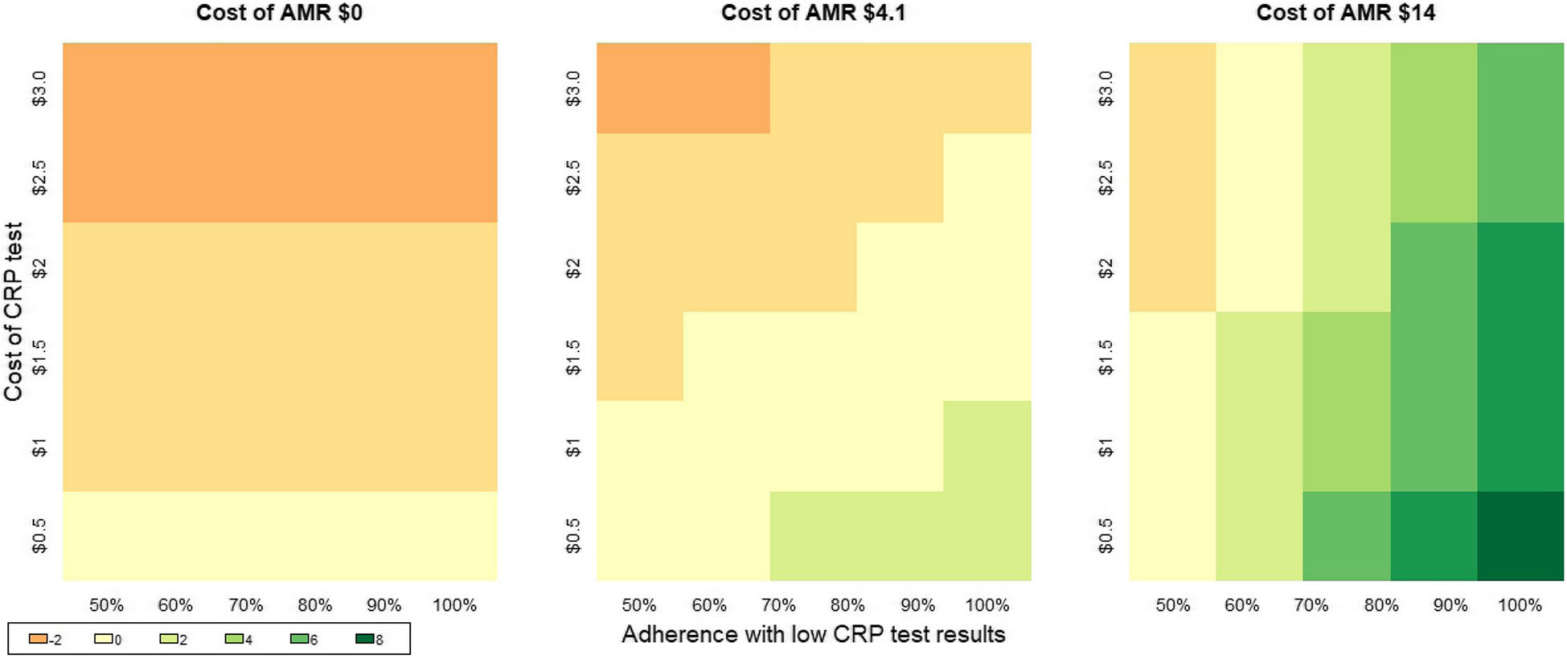
Net benefit for CRP testing by unit cost, test adherence, and cost of AMR. The 3 panels indicate the net-benefit of CRP testing in response to different configurations of the cost of the CRP test, the degree to which health workers adhere with the test results, and the economic cost of AMR per full course of antibiotic averted. The range of colours reflect the net-benefit of the CRP tests, with dark orange areas indicating instances where the use of the test is not cost-beneficial, and dark green areas where the test is most-cost-beneficial. With the exclusion of the costs of AMR ($0), a CRP test would be at best cost-neutral if it was low cost. With the inclusion of the costs of AMR, using either the baseline estimate of $4.1 or a higher estimate of $14, CRP testing would be cost-benficial even if the cost of the test was as high as $3, providing adherence with test results was high

With the additional assumed cost per CRP test of $1, the incremental cost per patient with the use of the test was $0.93. With a cost of AMR per full course of antibiotics of $4.1, the use of the test results in a negative net-monetary benefit, implying that with a 20 percentage point reduction in prescribing, the use of the test was not cost-beneficial. Figure 1 presents the sensitivity analysis for the net-benefit of the tests to their unit cost, the adherence to negative test results, and to the economic cost of AMR. This suggests that without consideration of the costs of AMR, the use of the tests is not cost-beneficial even with a low cost test and with high adherence to their results. With the inclusion of a cost of AMR of $4.1 per course of antibiotics, the test has a positive net-benefit if adherence exceeds 70% and the cost of the test is <=$0.5, or with adherence at 80% and a unit cost per test of $1. A higher cost of AMR of $14.1 per full course of antibiotics would imply a positive NMB so long as adherence exceeded 60%, even if the cost per test was as high as $3.

## Discussion

The trial demonstrated that CRP testing could safely reduce antibiotic prescription in primary health care settings. The primary cost data indicate that other than the cost of the test, there were no added direct medical costs and potential cost savings associated with the intervention. Assuming a unit cost per test of $1, the incremental cost per patient would be $0.93. With an estimated societal cost of AMR of $4.1 per course of a broad spectrum beta-lactam, the use of the tests would not appear to be cost-beneficial due to the limited adherence to their results. The sensitivity analysis, however, indicated that the tests could be cost-beneficial if healthcare workers refrained from prescribing to patients with low CRP at higher rates than observed in the trial.

### Implications of the study

The Wellcome Trust AMR Review estimated that by 2050 global economic losses due to AMR could accumulate to $124 trillion [24]. Who should be funding interventions to mitigate the spread of AMR is a challenging question. Patients or even policy makers in LMICs might not view this as a priority over interventions with more tangible short term benefits; this would imply limited and sub-optimal uptake from the global community perspective in its efforts to tackle AMR. The AMR Review appropriately highlighted the need for a global funding mechanism (‘*Diagnostic Market Stimulus pots*’), similar to the Global Fund for AIDS, Tuberculosis and Malaria, dedicated specifically to the development and scale-up of diagnostics and other interventions that safely reduce human antimicrobial consumption. This analysis suggests that investment of these resources in CRP testing in the current context could be cost-beneficial, providing adherence to their results is high.

The vast proportion of antibiotics dispensed for ARI through private pharmacies without prescription in Viet Nam needs to be properly considered [25]. Targeting the private sector could dramatically increase the impact of an intervention such as PoC CRP testing on unnecessary antibiotic use, but has major programmatic challenges. An obvious solution is to enforce the laws that are in place since 2006 prohibiting unregulated antibiotic sales, driving these patients to primary and community-care centres where they can be examined and tested. However, as indicated by the trial findings and this cost-benefit analysis, high adherence to the test is critical to ensuring their impact and cost-effectiveness. To achieve this, the introduction of CRP tests could be integrated into a broader public health campaign that includes training and education for both healthcare workers and patients, to maximize the benefits of both interventions and achieve a behavioural shift away from widespread use of antibiotics for minor complaints [26].

### Strengths and limitations

This analysis benefitted from detailed primary costing data from a large clinical trial in Viet Nam evaluating an intervention to address the urgent need for safe reductions in antibiotic prescribing in primary care. To our knowledge these are the only such data from an LMIC setting. Use of the economic costs of AMR averted in a cost-benefit analysis of the intervention also represents an advancement in our ability to perform economic evaluations of such interventions.

The study has numerous limitations. The primary measure of effectiveness in the trial was the proportion of antibiotic prescriptions averted but approximately one third of patients that were not prescribed an antibiotic went on to obtain them elsewhere. If the intervention were to be rolled out in routine care, this should be supplemented by further training and education for healthcare workers and patients to ensure better adherence to the test. Such programmes will incur higher costs, which were not accounted for in the analysis. The sensitivity analysis carried out here indicates that investment in such education and training programmes for patients and healthcare workers to improve adherence to CRP test results could itself be cost-effective.

The CRP test used in the trial relied on a quantitative reader, requiring some degree of technical experience, with a relatively high cost per test, whereas in the model we assume that lower cost lateral flow devices are used instead. It is possible that such devices would have a different impact on prescribing than that observed in the trial.

The methods used to quantify the costs of AMR averted due to reductions in prescribing have numerous limitations detailed in the paper describing their estimation [9]. In addition to these limitations, the adaptation of the costs of AMR calculated in the Thai setting to the Viet Nam context also assumed that Viet Nam and Thailand have similar epidemiological profiles for the prevalence of resistant infections which may not be the case.

As well as guiding whether or not to prescribe an antibiotic, CRP testing might also influence prescribing in terms of choice of antibiotic and duration of treatment, with implications for costs, health outcomes and AMR. As there was no evidence of this occurring in the trial this was not incorporated in the analysis.

Beyond the impact on AMR are other possible costs and health implications associated with CRP testing that were not accounted for. Adverse reactions occur in a small proportion of antibiotic courses, but the frequency of antibiotic use makes them account for approximately a quarter of all adverse events recorded in the hospital setting [27, 28]; a study of adverse drug reactions in emergency department visits found antibiotics to be implicated in a fifth of cases [29]. Second, while over-treatment is a challenge in all income settings, in many LMICs under-treatment of respiratory infection in patients with restricted access to antibiotics imposes a huge morbidity and mortality toll [30, 31]. In such settings CRP testing could therefore have a direct beneficial impact on health outcomes, through identification of patients that require antibiotic therapy and may not otherwise be detected [32].

## Conclusion

Use of CRP test in the context of primary care in LMICs is likely to incur a modest incremental cost, but this can be offset by the economic costs of AMR averted, providing adherence to their results is high. Whether patients or health providers in LMICs can and should shoulder the tangible costs is contestable; this study suggests that international donor support for these purposes is economically warranted. With low cost tests available and validated, large scale implementation of CRP point of care tests is feasible. Implementation of CRP testing on national scales will pave the way for novel and better systems that are currently cost-prohibitive once these are better validated and available at affordable price ranges.

## Additional file

Additional file 1: Primary costing data from a clinical trial of CRP guided treatment for respiratory illness in vietnamese primary care facilities. (DOCX 297 kb)

## Abbreviations

AMR: Antimicrobial resistance
ARI: Acute respiratory infections
CBA: Cost-benefit analysis
CRP: C reactive protein
GDP: Gross Domestic Product
LMIC: Low and middle income country
NHTD: National hospital for tropical diseases
NICE: National Institute for Health and Clinical Excellence
NMB: Net monetary benefit
PoC: Point of care
RDT: Rapid diagnostic test
US: United States
WHO: World Health Organization
