# Supplementary material for: C-reactive protein point of care testing in the management of acute respiratory infections in the Vietnamese primary healthcare setting – a cost benefit analysis
Source: Antimicrob Resist Infect Control. 2018 Oct 4;7:119. doi: 10.1186/s13756-018-0414-1 (PMC6172744; doi:10.1186/s13756-018-0414-1)
Supplement: Supplementary file 1 — Primary costing data from a clinical trial of CRP guided treatment for respiratory illness in vietnamese primary care facilities. (DOCX 297 kb) [file 13756_2018_414_MOESM1_ESM.docx]

**Additional file 1**

**C-Reactive Protein Point of Care Testing in the Management of Acute Respiratory Infections in the Vietnamese Primary Healthcare Setting – A Cost Benefit Analysis**

**Supplementary Material – direct and indirect costs for an episode of ARI in Vietnamese primary care settings**

**Patients and settings**

An open-label randomised controlled trial conducted in 10 primary healthcare centres in northern Viet Nam assessed the effectiveness of C reactive protein (CRP) point of care (PoC) testing in reducing inappropriate antibiotic use for patients with non-severe acute respiratory infection (ARI) (CONSORT diagram shown in Fig 1). Details of the study are described elsewhere.^1^ Briefly, 2,036 patients (1028 children, 1008 adults) were enrolled and received routine clinical examination and follow-up visit on day 4 (±1). Patients in the CRP group received additional evaluation by CRP PoC testing at both visits. Patients randomised to the control arm were treated according to routine practice based on clinical examination. Treatment for CRP patients was based on both clinical examination and test results, with different CRP cut-off values given for children aged 1-5 years (≤ 10mg/L) and those aged 6-60 years (≤ 20mg/L) to recommend withholding antibiotic prescription. The primary outcome was antibiotic use within 14-day follow-up. Secondary outcomes included the duration of symptoms, the frequency of re-consultations, serious adverse events, and reported patient satisfaction.

**Cost survey forms**

A survey was carried out to collect medical and non-medical costs related to the management of the illness on the day of enrolment (Annex 1). Medical costs consisted of those for examination, routine diagnostic tests (other than CRP testing, provided free of charge) and medications. Medical costs in Viet Nam are partially covered by health insurance and the remainder by patients’ out-of-pocket; both costs were recorded separately in the forms. The trial recruited outpatients only, therefore non-medical direct costs included those for transportation alone. At day 14 a similar form was used to collect any subsequent costs during the two-week follow-up period (Annex 1).

**Data analysis**

The data were cleaned and double entered into an electronic database and checked for quality by an independent data analyst. The analysis was based on the intention to treat (ITT) population including all randomised patients except for those who withdrew immediately, and analysis was according to the treatment arm. Medical cost data was summarized using mean and standard deviation (SD) regardless of skewed distribution. This is because means can be arithmetically manipulated the total healthcare cost of treating all patients, which is needed as the basis for medical policy decisions, but not median costs.^2,3^ Potential differences between the groups were compared by t-test for non-normal continuous data. P-values less than 0.05 were considered significant (2 tailed). The cost data were collected alongside the clinical trial from March 2014 to July 2015. All costs are reported in US Dollars ($) using the 2015 currency exchange rate from Viet Nam Dong (VND) of $1 = 22,500 VND.

**Costing study findings**

**Patient and provider costs on first attendance**

Of the 2036 patients enrolled, cost data were available for 100% of patients on Day 0. The total patients’ medical cost including costs for examination, diagnosis, and medicines on first attendance were not significantly different between arms (p=0.28). However, the cost for antibiotics were significantly lower in the CRP group (mean difference = -$0.18; 95% CI -$0.28 to -$0.08). This was slightly offset by a smaller increase in costs for other medication in the intervention arm (mean difference $0.10; 95%CI $0.03 to -$0.17) (Table 1). The cost for diagnosis was also significantly higher in the intervention group compared to control group. In both arms approximately 90% of the total medical costs were covered by health insurance.

**Patient and provider costs during the two weeks of follow-up**

Day 14 follow up costing data were available from 91% of enrolled patients (1849/2036); no statistically significant differences were found in demographic characteristics in patients with and without follow-up cost data. In the two weeks following first attendance, total medical costs were similar across treatment groups (p>0.05) (Table 2). The costs associated with subsequent antibiotics and hospitalization (included in the treatment cost) were not significantly different between arms (p>0.05).

In summary, there was no significant difference in household and provider costs in each of the trial arms, with a mean total cost from first attendance of $2.61 in the CRP group, higher but not significantly so than the mean cost of $2.47 in the control arm (p=0.28) and during two weeks of follow-up with $2.56 versus $2.33, respectively (p=0.57).

**Table S1. Summary of patient medical costs (in USD) by treatment arm on first attendance at 10 primary healthcare centres**

|  | **CRP (n=1017)**  **Mean (SD)** | **Control (n=1019)**  **Mean (SD)** | **Difference mean**  **(95%CI)** | **P value^$^** |
| --- | --- | --- | --- | --- |
| Total cost | 1.24 (1.53) | 1.31 (1.52) | -0.07 (-0.21 to 0.06) | p=0.28 |
| Examination cost   - Total - HI cover - Self-paid | 0.35 (0.62)  0.22 (0.60)  0.12 (0.29) | 0.34 (0.34)  0.20 (0.28)  0.14 (0.31) | 0.01 (-0.04 to 0.05)  0.02 (-0.02 to 0.06)  0·02 (-0.05 to 0.006) | p=0.84  p=0.24  p=0.14 |
| Diagnostic tests cost   - Total - HI cover - Self-paid | 0.18 (1.30)  0.15 (1.04)  0.04 (0.54)  0.18 (1.20) | 0.12 (0.85)  0.11 (0.73)  0.02 (0.16)  0.07 (0.86) | 0.06 (-0.04 to 0.15)  0.04 (-0.04 to 0.12)  0.02 (-0.01 to 0.05)  0.11 (-0.02 to 0.24) | p=0.23  p=0.33  p=0.26  p=0.11 |
| Antibiotic cost   - Total - HI cover - Self-paid | 0.51 (1.04)  0.48 (0.94)  0.03 (0.17) | 0.69 (1.23)  0.63 (1.04)  0.05 (0.49) | -0.18 (-0.28 to -0.08)  -0.15 (-0.24 to -0.07)  -0.02 (-0.05 to 0.01) | p=0.0004  p=0.0003  p=0.22 |
| Other medications   - Total - HI cover - Self-paid | 0.39 (0.63)  0.37 (0.60)  0.02 (0.09) | 0.33 (0.58)  0.32 (0.56)  0.01 (0.09) | 0.06 (0.01 to 0.12)  0.05 (0.01 to 0.11)  0.01 (-0.002 to 0.01) | p=0.02  p=0.03  p=0.13 |

**Table S2. Subsequent cost (in USD) by treatment arm during 14 days**

|  | **CRP (n=902)**  **Mean (SD)** | **Control (n=947)**  **Mean (SD)** | **Difference mean**  **(95%CI)** | **P value^$^** |
| --- | --- | --- | --- | --- |
| Total cost | 1.18 (1.37) | 1.28 (1.42) | -0.1 (-0.22 to 0.02) | p=0.12 |
| Treatment cost | 0.39 (0.63) | 0.33 (0.58) | 0.06 (0.01 to 0.12) | p=0.02 |
| Antibiotic cost | 0.18 (1.46) | 0.14 (1.14) | -0.04 (-0.07 to 0.16) | p=0.47 |
| Other medications cost | 0.07 (0.53) | 0.09 (1.12) | -0.02 (-0.09 to 0.06) | p=0.65 |

*^$^All comparisons were based on t-test.*

**Figure 1**: CONSORT diagram for the clinical trial


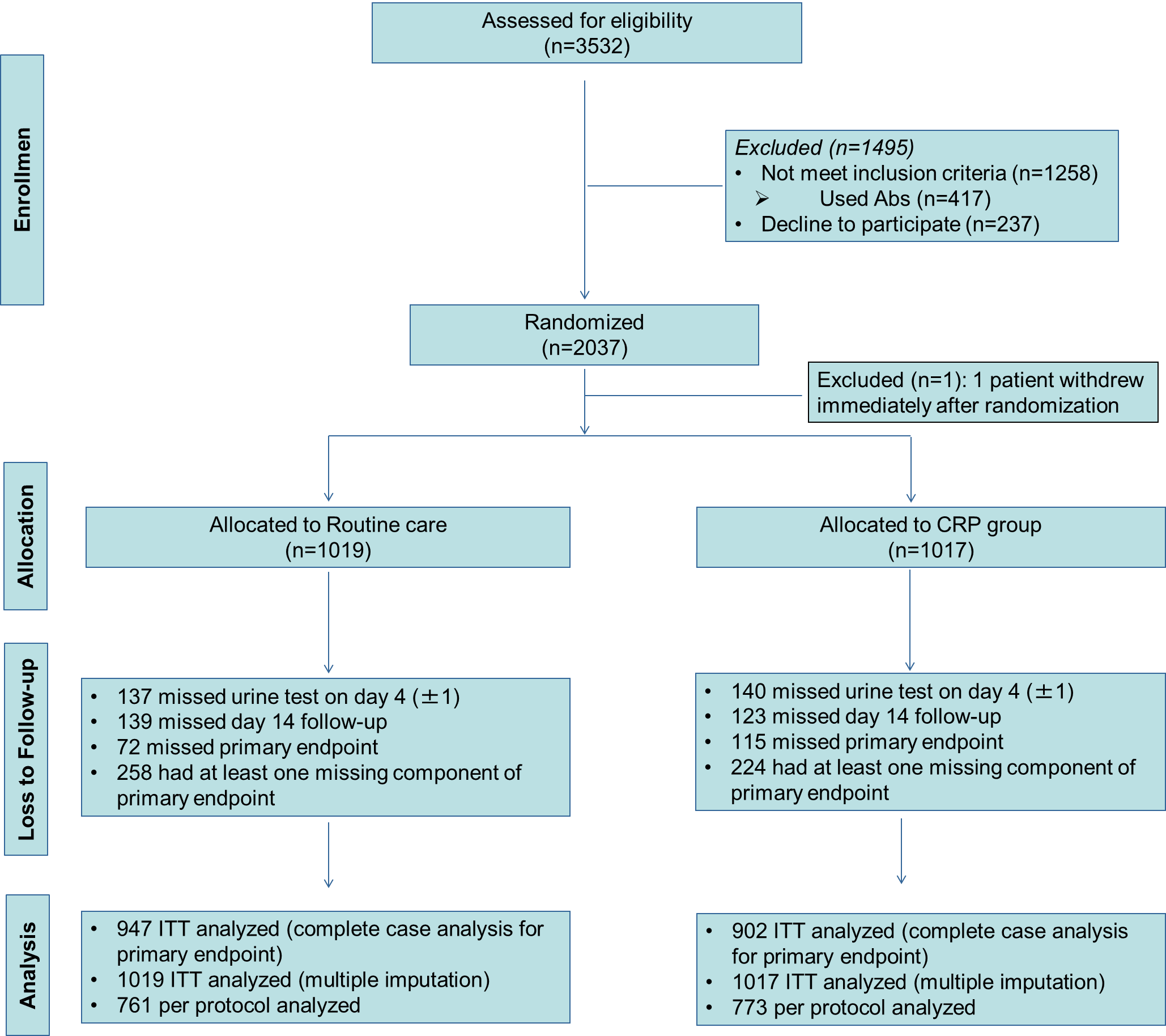


Excluded patients: age<1 or >65 years old: 244; severe respiratory infections: 16; hospital referral: 1; suspicion of tuberculosis: 3; liver disease: 6; medical history of neoplastic disease, congestive cardiac failure, COPD, insulin-dependent diabetes or renal disease: 110; pregnancy: 46; no access to telephone: 9; already taken antibiotics: 417; symptoms present for more than two weeks: 65; not able to come for follow-up visit: 169; decline to participate: 237; uncoded: 172.

**Annex 1. Cost survey on Day 0**

| **Cost survey on Day 0** |
| --- |
| **Patient Code: 05HN-[__\|__]-[__\|__\|__\|__] Patient initials: [__\|__\|__\|__\|__]** |
| 1. Informant ⭘ Patient ⭘ Mother ⭘ Father ⭘ Other ___________ |
| **PRE-CLINIC, TREATMENT & TRAVEL COST TO CLINICS (in vnd)** |
| 1. Before coming to the clinics today, what did you do to treat this illness? (Tick all that apply)   □ Nothing  □ Went to a clinic/hospital  □ Got drugs from pharmacy  □ Other, specify _________________ |
| 1. How much have you spent in treating this illness before coming to the clinics today, including costs of any medicines and any fees charged by providers?   Medicines fee: \|__\|__\|__\|-\|__\|__\|__\|  Examination fee: \|__\|__\|__\|-\|__\|__\|__\|  Diagnosis fee: \|__\|__\|__\|-\|__\|__\|__\|  Other: \|__\|__\|__\|-\|__\|__\|__\| |
| 1. How much money did you spend on transport to come to the clinics today?   \|__\|__\|__\|-\|__\|__\|__\| |
| **CLINICS DIAGNOSIS AND TREATMENT ON DAY 0** |
| 1. How much have you pay for examination fee today?   □ Cover by HI: \|__\|__\|__\|-\|__\|__\|__\|  □ Self-paid: \|__\|__\|__\|-\|__\|__\|__\| |
| 1. Have any diagnostic tests been performed at the clinics today?   Test Covered by HI Cost Self-paid Cost  □ None  □ X-ray □ \|__\|__\|__\|-\|__\|__\|__\| □ \|__\|__\|__\|-\|__\|__\|__\|  □ Blood test □ \|__\|__\|__\|-\|__\|__\|__\| □ \|__\|__\|__\|-\|__\|__\|__\|  □ Endoscopy □ \|__\|__\|__\|-\|__\|__\|__\| □ \|__\|__\|__\|-\|__\|__\|__\|  □ Other: □ \|__\|__\|__\|-\|__\|__\|__\| □ \|__\|__\|__\|-\|__\|__\|__\| |
| 1. Have any medications have been prescribed to you today?   Covered by HI Cost Self-purchased Cost  □ Nothing  □ Antibiotics □ \|__\|__\|__\|-\|__\|__\|__\| □ \|__\|__\|__\|-\|__\|__\|__\|  □ Cough preparations □ \|__\|__\|__\|-\|__\|__\|__\| □ \|__\|__\|__\|-\|__\|__\|__\|  □ Fever, pain killer □ \|__\|__\|__\|-\|__\|__\|__\| □ \|__\|__\|__\|-\|__\|__\|__\|  □ Anti-histamin □ \|__\|__\|__\|-\|__\|__\|__\| □ \|__\|__\|__\|-\|__\|__\|__\|  □ Corticoids □ \|__\|__\|__\|-\|__\|__\|__\| □ \|__\|__\|__\|-\|__\|__\|__\|  □ Vitamin □ \|__\|__\|__\|-\|__\|__\|__\| □ \|__\|__\|__\|-\|__\|__\|__\|  □ Other □ \|__\|__\|__\|-\|__\|__\|__\| □ \|__\|__\|__\|-\|__\|__\|__\| |

**Cost survey on Day 14**

| **Cost survey on Day 14** |
| --- |
| **Patient Code: 05HN-[__\|__]-[__\|__\|__\|__] Patients initials: [__\|__\|__\|__\|__]** |
| 1. Informant ⭘ Patient ⭘ Mother ⭘ Father ⭘ Other ___________ |
| **TREATMENT AND COURSE OF ILLNESS SINCE DAY 0 (in vnd)** |
| 1. Since leaving the health centre 14 days ago, what have you done to treat this illness, and how much have you spent including 2^nd^ visit?   □ Nothing  □ Went for 2nd study visit  □ Went to drug store  □ Returned to this clinics (except 2nd  □ Went to another clinics  □ Went to hospital  □ Hospital admission  □ Consulted traditional healer  □ Other, specify________________ |
| 1. For each option about, please specify the cost for   Transportation fee: \|__\|__\|__\|-\|__\|__\|__\|  Examination fee: \|__\|__\|__\|-\|__\|__\|__\|  Diagnosis fee: \|__\|__\|__\|-\|__\|__\|__\|  Medicine fee: \|__\|__\|__\|-\|__\|__\|__\|  Other: \|__\|__\|__\|-\|__\|__\|__\| |
| 1. Since leaving the health centre 14 days ago, what kind of medications you had to buy for this illness, and how much did they cost?   Cost (vnd)  □ Nothing  □ Antibiotics \|__\|__\|__\|-\|__\|__\|__\|  □ Cough preparations \|__\|__\|__\|-\|__\|__\|__\|  □ Fever, pain killer \|__\|__\|__\|-\|__\|__\|__\|  □ Vitamin \|__\|__\|__\|-\|__\|__\|__\|  □ Other \|__\|__\|__\|-\|__\|__\|__\| |

**Reference**

1. Nga DTT NT, Ninh Tran, Hung TM, Bich VTN, Long HB, et.al. Point-of-care C-reactive protein testing to reduce inappropriate use of antibiotics for acute respiratory infections in adults and children in the Vietnamese primary health care setting: a multi-centre randomised controlled trial. *The Lancet Global Health* 2016; **4**(9): e633-e41.

2. Thompson SG, Barber JA. How should cost data in pragmatic randomised trials be analysed? *BMJ* 2000; **320**(7243): 1197-200.

3. Briggs AH, Gray AM. Handling uncertainty in economic evaluations of healthcare interventions. *BMJ* 1999; **319**(7210): 635-8.
